# Chicago Medical Society

**Published:** 1883-09

**Authors:** 


### Society Reports.

### ARTICLE VII.

CHICAGO MEDICAL SOCIETY.

The Chicago Medical Society held a regular and well attended meeting June 18, 1883, in their club room in the Grand Pacific Hotel, with Dr. D. W. Graham in the chair.

After the usual routine of business, Dr. Henry Ogden presented a valuable paper on Obstetrics. This topic was made more interesting and prolific by a carefully written report on a "Trio of cases of Triplets," and exhibition of a placenta in one of the births.

Report published in full in this number.

A supplementary report was given, wherein Dr. J. W. Edwards of Mendota, had kindly furnished Dr. Ogden a case of triplets three girls, born to Irish parents on the morning of June 3, 1881, in that city; there were three separate placentæ in this case, and the children are now two years of age, and all living.

In the discussion, Dr. Mary H. Thompson said she had been much interested in the subject, and thought the paper a good one.

Dr. Henry VanBuren said he had attended 100 cases of labor before he had a case of twins, and attended 300 births when the case of triplets occurred as reported in the paper. He thought there was a peculiar throwing power in the fundus of the uterus in triplet births. The contractions of the uterus were peculiar, and different than in single births, and the cords were shorter.

Dr. H. D. Valin thought there some omissions in the paper that should have been further dwelt upon, especially regarding the history of the parent's parents.

Dr. C. W. Earle examined the case with Dr. VanBuren, in which there was but one placenta and three cords, and substantiated the report in the paper; he also reported a case occurring in the practice of Dr. White in 1879, in this city, where three male children were born, their aggregate weight being ten and a half pounds, but all died in three weeks of inanition; he thinks heredity has no influence regarding multiple births.

Dr. Houghton related a case occurring within his knowledge, of a woman giving birth to twins three times within the space of three and a half years.

Dr. J. H. Etheridge knew of a family in Canada, where in six confinements sixteen children were born, as follows: Four times triplets, and twice twins.

Dr. L. H. Montgomery knew a German family, residing near Shulsburg, Wis., where he formerly resided, and studied in his preceptor's office, in the summer of 1869. Dr. M. A. Fox then attended the man's wife, some five miles distant, and three boys were born. Just after the second boy was born, the woman fainted, and while in this state of syncope, the third child was born, when she recovered in a few moments, and upon being informed of the number, she expressed herself in Teutonic dialect, that, "dos ish kneuf." I visited the noble woman next day to see "if any of them had got away." She recovered rapidly, and so far as I know, the boys are yet living, and are about fourteen years of age.

Dr. E. Ingals, chairman of the committee appointed at the second previous meeting to confer with the Illinois State Board of Health relating to matters contained in the proceedings of the quarterly meeting of said Board, held in this city April 12, 1883, submitted the following report:

TO THE CHICAGO MEDICAL SOCIETY.

Your committee appointed to confer with the Illinois State Board of Health relating to matters contained in the proceedings of the quarterly meeting of said Board, held in Chicago, April 12, 1883, ask leave to submit the following report:

We desire to acknowlege the courtesy of the Board in furnishing us, from its official correspondence, all the information we desired relating to the subjects under consideration. The following from Secretary Rauch's report to the Board, could not escape the attention of the profession, and seems to call for some action on its part:

Proofs are on file that students are graduated without having studied the required length of time, or without having studied under a preceptor; who have attended only one course of lectures; who have attended two courses in one year, without the necessary reading period intervening; who were not 21 years of age at the time of graduation, and who, in general, are not at all competent to practice medicine.

While preparing this portion of my report, the following case in point presents itself. An official proceeding requires that Dr. ——, a graduate of one of the most popular and widely-known colleges in the country, detail his acquirements in pharmacy. He is asked what experience he has had in compounding medicines, and replies that he has had none.

"Did you not put up prescriptions under your preceptor while a student?"

"No, sir; I didn't have any preceptor."

"Why, I supposed that medical colleges required that their graduates should have read or studied medicine under a preceptor for three years. How did you get through? How did you graduate?"

"Well, I attended two courses of lectures, paid the fees and got my diploma."

An examination of the files in the secretary's office resulted in finding the following communication from Dr. ——, received May 20, 1882:

————————, ————, Ill.

To the *Secratary* State *boar* of health *Deear* Sir I sent you my *dipluma* early last March and have not head from it *sinc* did you receive it or do you know anything about it I am becoming quite anxious concerning its *safty* My *dipluma* is from——— Medical College——— ——dated ————1882 I also sent you a letter containing a one dollar bill to pay for the *certifficate* If you will give me the information I *requist* I shall be greatly obliged to you.

Yours very respectfully

——————————— M.D.

In the annual announcement of the college which issued this diploma, among the regular requirements for graduation one is stated to be "such primary education as is clearly requisite for a proper standing with the public and the profession;" and another that "he must have pursued the study of medicine three years." That the former requirement was ignored is obvious from the letter quoted; and it is probably doing no one injustice to accept Dr. ——— statement—that he "attended two courses of lectures, paid the fees and got a diploma," as full summary of his medical education, so far as the college was concerned.

As a result of my own official experience during the past six years, I think it entirely within bounds to say that a strict adherence to the advertised requirements is the exception among colleges rather than the rule. In fully three-fourths of those which have come under my observation, there have been irregularities of more or less gravity.

The charges that "students are graduated without having studied the required length of time, or without having studied under a preceptor; who have attended only one course of lectures; who have attended two courses in one year, without the necessary reading period intervening; who were not twenty-one years of age at the time of graduation, and who, in general, are not at all competent to practice medicine, are based on correspondence on file in the office of the Board, and on other evidence that has come to its knowledge. On first reading Dr. Rauch's report, we suspected that the Board had been imposed upon in the matter of the letter above quoted, and that it was spurious; for we did not believe that after the subject of a higher medical education had been as thoroughly discussed as it has been for a number of years, and the judgment of the profession in its favor was so pronounced and well understood, that any recognized, popular and widely-known college would, only one year since, confer the degree of Doctor of Medicine on a person who would write this shameful letter. The history of this transaction is as follows: This graduate forwarded to the Board of Health his diploma, and with it his affidavit that he was the person on whom the degree was conferred. The diploma and affidavit would entitle him, under the rules of the Board, to receive without question his license to practice in Illinois. To his communication he neglected to append his address, and consequently he received no reply, and this circumstance in due time called out the above letter. Now, the signature to this letter and that subscribed to the affidavit, we know from personal inspection to be by the same hand. According to our statute and ethical laws this graduate is entitled to every right and privilege that is accorded to the most accomplished practitioner in the State, and only the accident of the omitted address brought the transaction to public notice. The following form, rendered in our own vernacular tongue, embraces the substance of the diploma issued by all of our medical schools:

"Whereas, it is an established usage to confer academical degrees on those whose character and knowledge entitle them to respect and confidence; know ye that ——— ———, having complied with the requirements of our College, and given ample evidence of his learning and skill, we have by authority of the State of ——— conferred on him the degree of Doctor of Medicine, together with all the rights and privileges thereunto belonging. In testimony whereof, we have granted this Diploma, signed with our hands, and sealed with the seal of our college." It is a sad reflection, that a man who has misspelled above ten per cent. of the words of this simple letter above quoted, holds a diploma, signed by the faculty and officers of a recognized medical college, as proof that "he has complied with its requirements and given ample evidence of his learning and skill." We have the authority of a member of our State Board of Health for saying, that there are on file in its office not less than two hundred instances of graduates in medicine who hold this dubious evidence of learning, who cannot spell diploma. We have been furnished from this source with seven different and incorrect ways of spelling this word of seven letters. They are as follows: diaploma, diplomy, diplomer, diplomah, diaplemy, diapluma, diplama. It seems a marvel that these ingenious orthographers have all agreed to commence the word with D. The sad comedy of this subject is worthy of the pencil of a Hogarth. Fancy the Alma Maters of these two hundred ignorant persons, solemnly conferring on each of them the Degree of Doctor of Medicine, in consideration of "his having complied with the requirements of our college, and given ample evidence of his learning and skill." But when we think of such a person at the bedside of the sick, clothed in professional authority, the scene suggests a delineator of tragedy rather than comedy. We have no direct means of testing the medical attainments of these two hundred graduates; but every one knows that it is impossible for such uninstructed minds to acquire in any degree the knowledge essential to a decent discharge of the duties of a practitioner of medicine. All will admit that the practice of conferring degrees on such illiterate persons by our institutions of learning should be stopped; but it is not quite clear what methods are best for accomplishing this result. The efforts of the State Board "to secure one common Examining Board on preliminary education for the six medical colleges of Chicago" is to be commended. The secretary of the Board has communicated with all the medical colleges of this city on the subject, and a number of them have given the proposition their approval, and expressed a willingness to co-operate in carrying the purpose into effect. The rule now adopted by our State Board, of not recognizing any college as in good standing that does not require of the student evidence of the possession of a fair degree of primary education before he is permitted to matriculate, if honestly complied with by the colleges, would keep all these persons who cannot spell diploma out of the profession, but to cover all contingencies, it would be safer for the sick to allow none to practice without a license, and to license none but graduates who had passed a satisfactory examination before a board constituted for the purpose, which board should be independent of the schools that confer the degree of Doctor of Medicine. Our Board of Health should freely exercise the right it has to refuse to recognize diplomas of schools of doubtful character, and to revoke the certificates of all practitioners who clearly convict themselves of being unworthy to retain them.

Our State Board of Health commends itself to the confidence of the public to the extent of the acquirements which it expects of non-graduates, who apply to it for a license to practice, as shown by the printed list of questions submitted for answer to the applicants who presented themselves for examination at its last meeting. The standard required was not too high, but if it is maintained it will drive applicants to the colleges for a degree, as an easier way of entering the profession than to go before the State Board for an examination. No license to practice was issued to non graduates at this meeting, as none of the eighteen applicants could correctly answer eighty per cent. of the questions given. The public more than the profession are interested in a proper solution of these questions.

(Signed,)

E. INGALS,

R. G. BOGUE,

A. H. FOSTER, } *Committee.*

After its reading, Dr. S. Strausser moved that the report of the committee be received and the gentlemen discharged ; seconded by Dr. J. H. Etheridge, and the motion was carried.

Dr. R. E. Starkweather thought the information contained in the report should be more extensively promulgated, and moved that the secretary be authorized to have printed 500 copies, and distributed to the members and others in the profession. The motion was discussed at some length, but did not receive a second. Dr. Earle thought our salvation need not lie in the State Board of Health, especially regarding their views on spelling, old doctors—many of them—could not spell good, but were splendid practitioners.

Dr. J. A. Robison quoted the remarks he had heard at the recent meeting of the State Society, by a member there, who said, " Students that go into perceptor's offices should be better educated, and to get at the bottom of this important subject, perceptors should require of a student more thorough preliminary education before entering the study of medicine.

Dr. Ethridge argued at length upon the subject of Medical

Education, and a preliminary examination of students. He thought but very few doctors anywhere could answer 50 per cent. of the questions pertaining to the 998 medicines in the works on therapeutics.

Dr. Starkweather also spoke of the merits a student had to possess before he would be admitted in Cambridge college or the Oxford school, England ; and then the Society adjourned for two weeks.

L. H. M.

---

P. S.—Since the paper in the first part of this report was read a woman on Emma street, in the north west portion of the City has given birth to three children. All were alive and are doing well, along with the mother.

L. H. M.

---

